# Health Care Workers’ Experience With a Psychological Self-Monitoring App During the COVID-19 Pandemic: Mixed Methods Study

**DOI:** 10.2196/70412

**Published:** 2025-08-07

**Authors:** Lydia Khaldoun, Francois Bellemare, Christine Genest, Nicolas Bergeron, Steve Geoffrion

**Affiliations:** 1 Centre de recherche de l'Institut universitaire en santé mentale de Montréal Montreal, QC Canada; 2 École de psychoéducation Faculté des arts et des sciences Université de Montréal Montreal, QC Canada; 3 Département de psychologie Faculté des arts et des sciences Université de Montréal Montreal, QC Canada; 4 Faculté des sciences infirmières de l'Université de Montréal Université de Montréal Montreal, QC Canada; 5 Centre de recherche du Centre hospitalier de l'Université de Montréal Montreal, QC Canada; 6 Département de psychiatrie et d'addictologie Faculté de médecine Université de Montréal Montreal, QC Canada

**Keywords:** psychological self-monitoring, self-monitoring app, adherence, user experience, health care workers, well-being

## Abstract

**Background:**

Health care workers (HCWs) are at risk of experiencing psychological distress, particularly during the COVID-19 pandemic. Psychological self-monitoring apps may contribute to reducing symptoms of depression, anxiety, and trauma exposure by enhancing emotional self-awareness. This study focused on how a basic psychological self-monitoring app was experienced by HCWs during the COVID-19 pandemic in Quebec by exploring users’ experience and factors contributing to their adherence.

**Objective:**

This study aimed to explore HCWs’ experiences with a psychological self-monitoring app, including if their satisfaction with the app, their perception of its contribution to self-awareness, and their experience of distress influenced their adherence to the app.

**Methods:**

HCWs in Quebec were invited to respond weekly to questions about their well-being via a mobile app. A convergent mixed methods design was used. Sample data (N=424) were collected from the app, a postparticipation questionnaire was administered, and 30 semistructured interviews were conducted. Correlations and hierarchical multiple regression models were conducted to examine possible factors influencing participants’ adherence, and a thematic analysis was used to further explore their experience.

**Results:**

Over a 12-week-period, mean adherence to the psychological self-monitoring app was 74.5% (SD 29.4%) and mean satisfaction was 80% (SD 20%). Most participants perceived that the app contributed moderately (165/418, 39.5%) or a lot (140/418, 33.5%) to enhancing their self-awareness. The significant regression model (*F*_5,401_=6.59; *P*<.001) suggested that around 7.6% of adherence variation could be explained by satisfaction (β=.16; *t*_401_=3.14; *P*=.002) and the app’s perceived contribution to self-awareness (β=.15; *t*_401_=2.88; *P*=.004). Biological sex (369/419, 88.1% female and 50/419, 11.9% male), age (mean 40.8, SD 9.9 y), and the experience of psychological distress at least once in 12 weeks (228/420, 54.3%) were not statistically significant predictors of adherence. Emergent themes from the 30 interviews highlighted participants’ experiences. Psychological self-monitoring was seen as an introspective practice, with reports of enhanced self-awareness and self-care practices. Interviewees generally considered the app as practical, but it did not suit everyone’s preferences. Potential app enhancements were provided by the participants.

**Conclusions:**

A simple psychological self-monitoring app could be an interesting tool for HCWs who wish to improve their self-awareness and prevent psychological distress, particularly in health crises such as pandemics.

## Introduction

### Background

Workers in health and social services networks (health care workers; HCWs), are exposed to various stressful occupational situations [1,2]. They are reported to be prone to psychological distress [3,4], characterized by painful mental and physical symptoms often assessed through self-reported measures of posttraumatic stress (PTS), anxiety, and depression [5]. Furthermore, times of crisis such as the COVID-19 pandemic can be particularly distressful for HCWs [6]. A recent meta-analysis reported that the prevalence of depression and anxiety among HCWs was 37% and 39%, respectively [7], while another found that 47% of the HCWs experienced burnout, 38% experienced anxiety, 34% experienced depression, 30% experienced acute stress disorder, and 26% experienced PTS disorder [8].

Exploring and assessing efficient ways to support HCWs is crucial. On the basis of the National Institute for Health and Care Excellence recommendations [9], a continuous monitoring of various reactions could be an effective strategy for identifying individuals at risk and preventing the development of mental health disorders. Hence, psychological self-monitoring can be defined as the examination of one’s personal condition through one’s thoughts, emotions, behaviors, and consequences.

Studies suggest that psychological self-monitoring may promote emotional self-awareness, which is the ability to recognize and describe one’s mood [10]. For example, in a qualitative study (N=21) tracking mood and physical activity in participants with bipolar disorder, half of the participants reported that psychological self-monitoring helped them recognize their emotional states and link them to factors such as sleep or exercise, thereby promoting behavioral changes [11]. Thereon, researchers have found that emotional self-awareness plays a mediating role in the relationship between psychological self-monitoring and psychological distress reduction [12-16]. Subsequently, awareness of a state of stress would lead the person to use coping strategies to adapt and cope with a situation or an environment [17]. Emotional self-awareness has been identified as an important factor for the well-being of prospective HCWs [18]. Hence, fostering emotional self-awareness with psychological self-monitoring might reduce psychological distress among HCWs, thereby improving their well-being.

To facilitate psychological self-monitoring, using digital technology is a promising solution, particularly during a pandemic [19,20]. Reviews have shown that mobile devices can improve users’ psychological well-being even without professional guidance [21-24]. Notably, apps that reduce psychological distress symptoms often invite users to practice psychological self-monitoring [12,25-28]. However, those apps offer other components and features, such as behavioral interventions and educational modules. There is limited understanding of psychological self-monitoring’s isolated influence on mental health management, and no studies previously focused on HCWs.

In addition, a major issue raised by researchers is the actual use of a digital tool. In a systematic review of available digital self-help interventions, indications of sustained use, such as the completion of all assessments or the use of the apps for more than 6 weeks, varied from 0.5% to 28.6% [29]. In this regard, adherence is the term often used to describe how much a technology is used [30] and is mostly measured by the number of logins, completed modules, or completed activities [31]. Measures of adherence have been positively associated with positive psychological outcomes in digital interventions [31]. A younger age and a biologically female sex were found to positively influence adherence [32,33]; however, research is needed to identify other factors [34].

Exploring users’ experience could provide a holistic perspective on their interaction with the psychological self-monitoring app. User experience reflects the perceptions and responses resulting from the use of a digital tool [35]. It can be investigated by analyzing induced feelings, judgments, and behaviors [36], and both qualitative and quantitative approaches can be used.

In parallel, the expectation-confirmation model suggests that app users form expectations, and their satisfaction is determined by how well those expectations match their actual experience [37]. In this context, satisfaction could be conceptualized as an overall evaluation of user experience with the psychological self-monitoring app [38]. Considering that satisfied users are more likely to sustain app use [32,37,39,40], a positive association could be hypothesized between adherence and satisfaction with a psychological self-monitoring app. According to the same model, perceived usefulness is proposed to be another key factor influencing adherence [37], a finding supported by reviews [32,33,41]. Indeed, users who feel that a psychological self-monitoring app positively influences their psychological state or their emotional self-awareness may perceive the app as useful, and thus, have higher adherence. However, the experience of psychological distress may or may not influence adherence, as conflicting results are found in the literature [34,42-47]. In a psychological self-monitoring app, a higher adherence could possibly be expected from users who experienced psychological distress, as they might have felt a greater need to monitor their psychological state.

### Objectives

In short, studies suggest that a psychological self-monitoring app could help users become more aware of their emotional state and could lead to improvements in well-being management. Further exploration is needed to discover the user experience with a simple psychological self-monitoring app and potential factors associated with its adherence. This study investigated Canadian HCWs’ use of a basic psychological self-monitoring app during the first 2 COVID-19 waves in Quebec [48-50]. The first objective was to examine whether adherence to a basic psychological self-monitoring app was associated with participants’ satisfaction, perceived app contribution to enhance emotional self-awareness, and the presence of psychological distress, when adjusting for age and biological sex. It was hypothesized that adherence would be higher whenever participants (1) had an increased satisfaction, (2) had an increased perception of the app’s contribution to emotional self-awareness, and (3) experienced psychological distress during their self-monitoring. The second objective was to explore user experience through thematic analysis of qualitative data.

## Methods

### Study Design

Considering the exploratory aims of this research, a convergent mixed methods design was adopted [51]. Quantitative data were collected from a psychological self-monitoring app and an online questionnaire. Qualitative data were collected from individual interviews.

### Study Population

Participants were Canadian HCWs mostly working in urban areas of Quebec’s integrated health and social services centers. They had participated in a program implemented during the COVID-19 pandemic to monitor HCWs’ psychological distress with an app. Inclusion criteria were having access to a smartphone and to Wi-Fi and working during the participation period in 1 of the 8 following integrated health and social services centers: University of Montreal Hospital Centre, Est de Montréal, Centre-Sud de Montréal, de-la-Capitale, Montérégie, Laval, Des Îles, and Estrie-CHUS. There were no exclusion criteria as all HCWs in these centers were invited to participate. Convenience sampling was used and participation to each phase of the project was voluntary. For the first objective, 424 users (n=369, 87% women; n=50, 11.8% men; and n=5, 1.2% missing data) accepted to fill out a postparticipation questionnaire. Participants’ age ranged from 18 to 68 (mean 40.8, SD 9.9) years and their years of work experience ranged from 0 to 43 (mean 13.6, SD 9.6) years. For the second objective, the analyzed data were collected from a subsample of 25 women and 5 men who were randomly selected for an interview. Their age ranged between 24 and 57 (mean 43, SD 9) years, with 0 to 30 years of work experience (mean 15, SD 9 years).

### Ethical Considerations

Ethical approval for the research project involving the self-monitoring app was granted by the University of Montreal Hospital Centre research ethics committee (reference: MP-02-2021-8963). Approval for this secondary analysis was granted by the University of Montreal’s research ethics committee in education and psychology (reference: 2023-4981). Informed consent was obtained from all individual participants included in the study. While participants did not receive monetary compensation, they were eligible to win 1 of 40 Can $50 (US $36.35) prepaid cards. All study data were deidentified and identifying details were removed from verbatims to ensure confidentiality.

### Procedure

Between May 2020 and February 2021, 831 HCWs voluntarily participated in a psychological distress self-monitoring program. Following methodological aspects of the ecological momentary assessment [52], participants were asked to answer a series of questions on an app each week over a 12-week period. They installed the Avicenna app, formally known as Ethica, a platform that enables researchers to collect data provided by their participants [53]. The app sent weekly notification prompting self-monitoring with subsequent reminders every 12 hours. Data collection was confidential.

**Figure 1:**
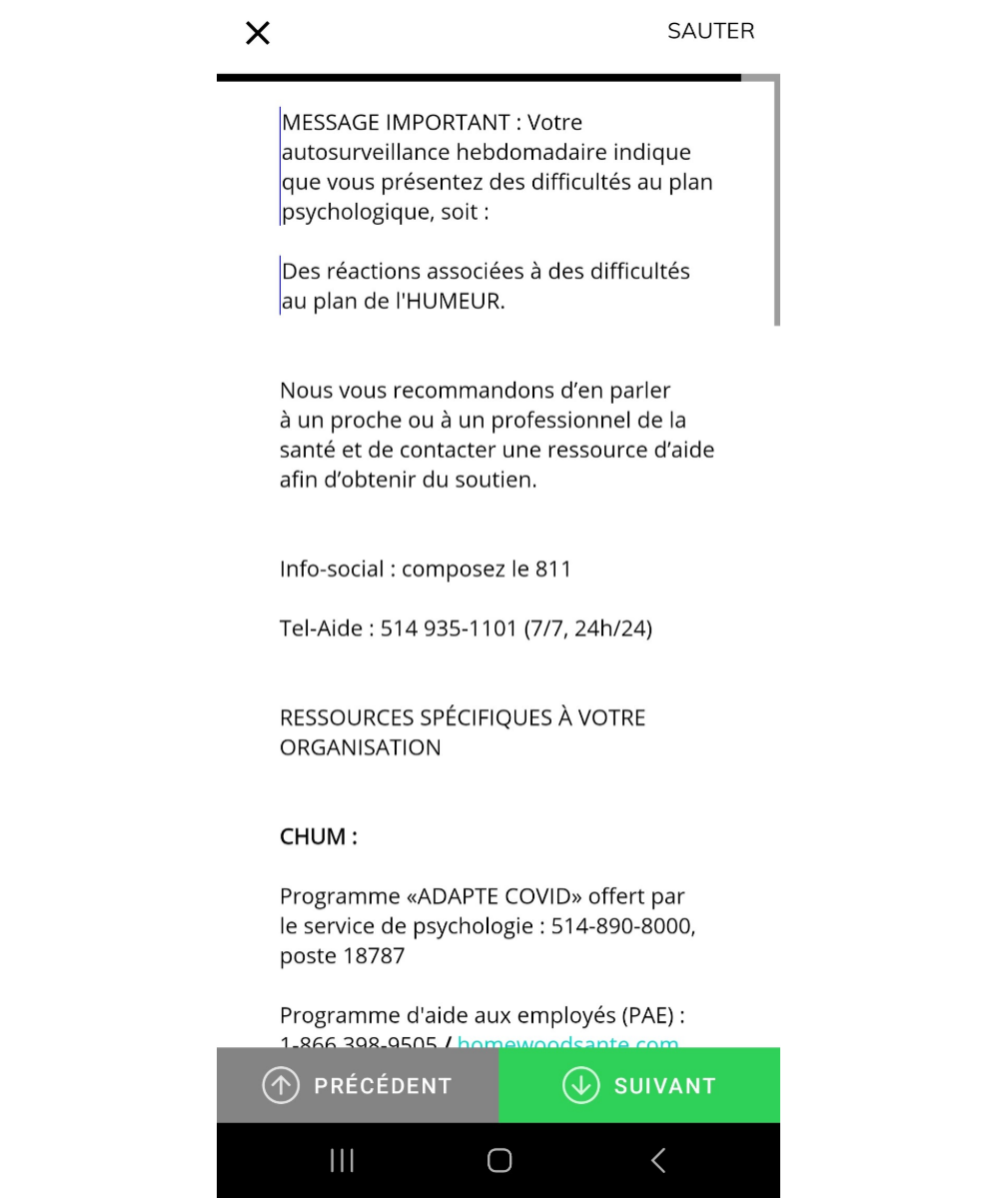
Screenshot of the psychological self-monitoring app in case of distress.

On the app, the questions assessed several elements related to COVID-19 exposure, perceived availability of social support, and perceived quality of life (Multimedia Appendix 1). In addition, anxiety, PTS, and depression symptoms were measured by validated questionnaires (see the Instruments and Measures section). When a participant’s self-reported responses reached the significant threshold of one or more of these 3 validated questionnaires, the participant received a message indicating the presence of significant distress, followed by a list of available resources (Figure 1). If no significant distress was detected, only the list of resources was displayed. In the event of suicidal ideations, the regional helpline’s contacts details were displayed on the next page. In addition, an email was automatically sent to the project coordinator, who ensured a follow-up call with the participant.

After the longitudinal data collection phase, between February and April 2021, users were invited to complete a postparticipation questionnaire hosted on the SurveyMonkey site [54]. Multiple-choice questions were asked to assess user experience with the psychological self-monitoring app, including perceived satisfaction and perceived contribution of the app to self-awareness (Multimedia Appendix 2). Formal psychometric validation was not conducted before deployment. However, to support content validity and face validity, items were designed based on existing frameworks for evaluating digital tools and were reviewed by the multidisciplinary researchers for clarity and relevance. Out of the 194 respondents interested in a telephone interview to further explore their experience, 30 were randomly selected. Interviews were individual, led by the research coordinator and lasted 15 minutes on average. They were transcribed by members of the research team, who ensured confidentiality by removing any data that could identify specific individuals.

### Instruments and Measures

#### Outcome: Adherence to the Psychological Self-Monitoring App

Adherence was measured using each participant’s completion rate. Completion rate was calculated by considering the frequency of completed weekly monitoring over 12 weeks. An adherence rate of 100% meant that the participant used the app 12 times, as intended.

#### Association 1: Satisfaction

Satisfaction was measured in the postparticipation questionnaire, which was specifically developed for this study. Participants rated their satisfaction with the app used for psychological self-monitoring (eg, ease of installing the app and user-friendliness of the app) on a scale between 0 and 10, which represented “extremely dissatisfied” and “very satisfied,” respectively.

#### Association 2: Perceived Contribution of the App to Self-Awareness

Perceived contribution to self-awareness was also measured in the postparticipation questionnaire. Participants rated the extent to which psychological self-monitoring on the app raised awareness or prompted reflection about their well-being. Responses varied according to a 4-point Likert scale, corresponding to 0=“not at all,” 1=“a little,” 2=“moderately,” and 3=“a lot.”

#### Association 3: Experience of Psychological Distress

Psychological distress was measured by considering 3 validated psychometric tests that participants weekly filled in the psychological self-monitoring app. During psychological self-monitoring, if at least one score reached the threshold for at least one of these questionnaires, significant psychological distress was identified. This variable was coded either as 0=“absent” or 1=“present.”

The Generalized Anxiety Disorder-7 [55] is a 7-item questionnaire assessing general anxiety symptoms. Participants indicated on a 4-point Likert scale (0=“not at all,” 1=“several days,” 2=“more than half the days,” and 3=“nearly every day”) how often they have been bothered over the past week by each symptom. An example of an item is “feeling nervous, anxious or on edge.” The total score ranges from 0 to 21 and was calculated by adding up the scores for all 7 items. A total score of ≥10 (cut-off) indicated moderate or severe generalized anxiety symptoms. In this study, participants filled the French version of the questionnaire weekly. It has been validated with an excellent internal consistency (Cronbach α=0.90) [56].

**Figure 2:**
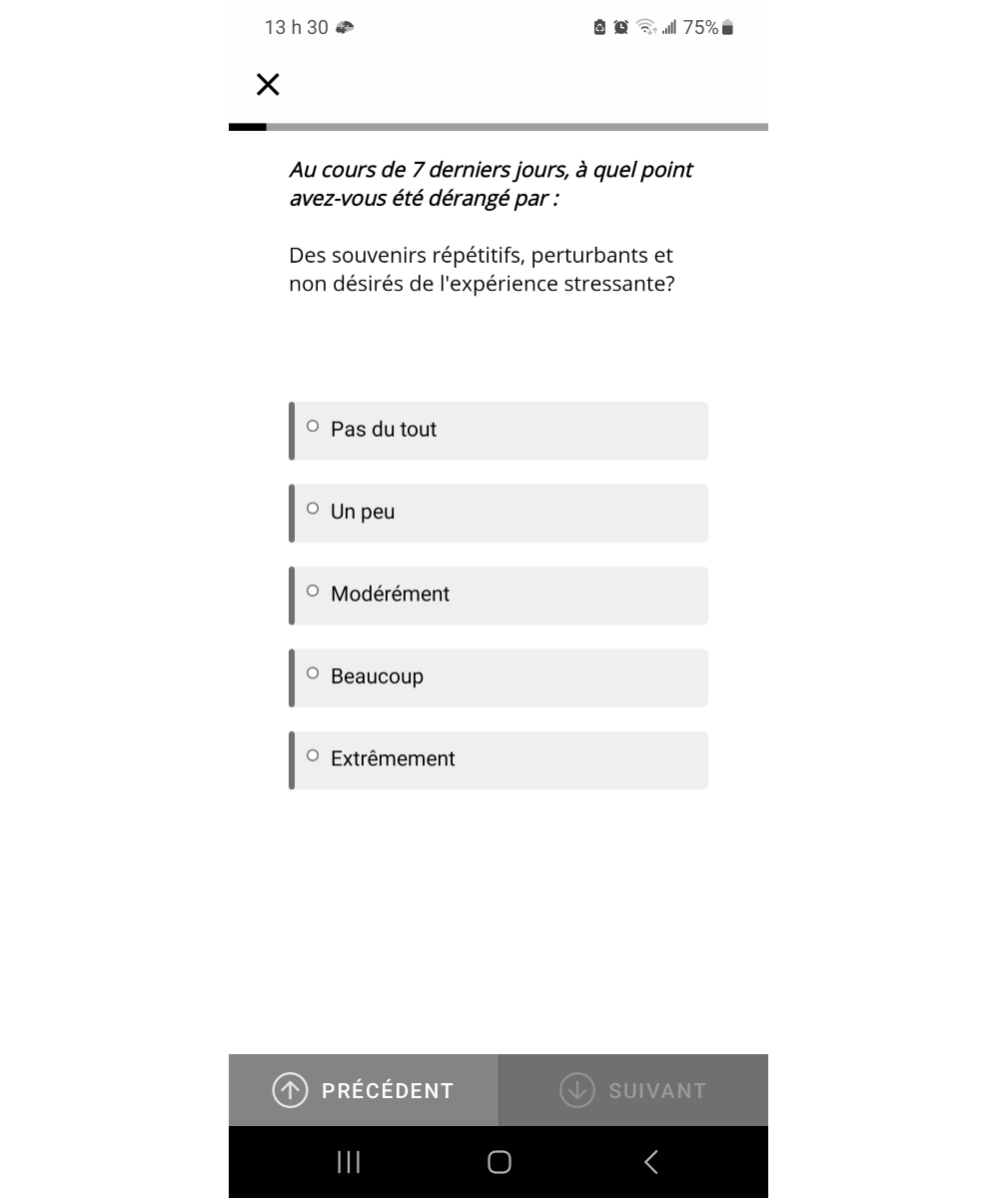
Screenshot of the psychological self-monitoring app assessing posttraumatic stress symptoms.

The PTSD Checklist for DSM-5 [57] is a self-report questionnaire assessing the severity of PTS symptoms. Participants indicated how bothered they were over the past week by each symptom on a 5-point Likert scale (0=“not at all,” 1=“a little bit,” 2=“moderately,” 3=“quite a bit,” and 4=“extremely”). An example of an item is “repeated, disturbing, and unwanted memories of the stressful experience” (Figure 2). A total score was calculated by adding up the scores for all items and the cut-off was set at 13. In this study, participants filled an abbreviated (8 items) French version, which was previously validated [58,59] with a sensitivity of 0.9, a specificity of 0.23, and a Cronbach α of 0.87.

The Patient Health Questionnaire-9 (PHQ-9) [60] is a 9-item questionnaire assessing depressive symptoms. Participants indicated how much they have been bothered over the past week by each symptom on a 4-point Likert scale (0=“not at all,” 1=“several days,” 2=“more than half the days,” and 3=“nearly every day”). An example of an item is “little interest or pleasure in doing things.” A total score was calculated by adding up the scores for all items, and the cut-off was set at 10. In this study, participants filled the French version, which was previously validated with a Cronbach α of 0.86 [61,62].

#### User Experience

To address the second objective, individual telephone interviews were conducted. These semistructured interviews followed a grid (Multimedia Appendix 3). Its development was inspired by the Mobile App Rating Scale, a reliable tool for classifying and assessing the quality of mobile health apps [63]. It was adjusted to align with the research objectives, exploring subjects such as experience and satisfaction with the app, psychological self-monitoring, emotional self-awareness, coping strategies, and app use.

### Data Analysis

#### Statistical Analysis

SPSS Statistics (version 29.0.1.0; IBM Corp) was used to carry out the analysis. Missing data were not statistically addressed considering they composed less than 5% of each examined variable. Statistics were computed based on available data for each variable.

First, descriptive statistics were used to examine frequency, average, and SD for each variable. Next, bivariate correlations explored relationships among biological sex, age, app adherence, satisfaction, perceived contribution to self-awareness, and psychological distress, with *P*<.05 considered statistically significant. Correlation strengths were classified as small (*r*≈0.10), medium (*r*≈0.30), and large (*r*≥0.50) [64]. Finally, a hierarchical multiple regression was conducted to further examine whether satisfaction, perceived contribution to self-awareness, and psychological distress influenced adherence to the psychological self-monitoring app. Confounding variables were participants’ biological sex and age.

During the review processes, nonparametric tests were conducted to examine whether there were significant differences between app users who did not participate in either the questionnaire or the interview, those who only participated in the questionnaire, and those who participated in both the questionnaire and the interview. Subsequently, inconsistencies were identified in the coding of psychological distress, specifically regarding the cut-off score used for the PHQ-9. While a cut-off score of ≥10 is widely accepted for identifying major depression [60], some data had been coded using a threshold of ≥9. To address this issue, a consistent cut-off score of ≥10 was used for PHQ-9, a rigorous reprocessing and verification of all study variables were done, and all analyses were carefully rerun to ensure the accuracy and reliability of results.

#### Thematic Analysis

An inductive and progressive thematic analysis was conducted using Braun and Clake approach [65] to analyze data from 30 interviews. Analysis began with a familiarization with the material, followed by generating initial codes for significant excerpts aligned with the study’s aims. These codes were reviewed to ensure they reflected participants’ statements. Next, codes were compiled into a separate file for sorting into potential themes, grouping salient units into progressively named and refined themes. Patterns within each theme were organized into subthemes, and an interjudge agreement was sought between first and second author to enhance clarity. Results were documented in a table with central themes, subthemes, codes, and verbatim excerpts, culminating in a thematic map summarizing the findings. In this paper, fictive names were attributed to participants.

## Results

### Sample Analysis

Out of the 831 app users, 407 (49%) did not complete the postparticipation questionnaire, while 424 (51%) did, including 30 (3.6%) participants who also took part in an interview. The study sample consisted of users who completed the questionnaire, including those interviewed. Descriptive statistics for this study sample are presented in Table 1. To assess potential selection bias, users who did not complete the postparticipation questionnaire were compared with the study sample (Table S1 in Multimedia Appendix 4). Results of Mann-Whitney *U* and chi-square tests indicated no significant differences in biological sex (*P*=.58) or psychological distress (*P*=.77). However, study sample was on average older (*U*=74,310; *P*=.02; *r*=−0.08) and had significantly higher adherence scores (*U*=54,513; *P*<.001; *r*=0.33) than nonrespondents. In addition, participants who were interviewed were compared to those who only completed the questionnaire (Table S2 in Multimedia Appendix 4). Results of Mann-Whitney *U* and chi-square tests indicated no significant differences in age (*P*=.15), biological sex (*P*=.41), satisfaction (*P*=.56), psychological distress (*P*=.96), or perceived contribution to self-awareness (*P*=.25). However, interviewees had significantly higher adherence scores (*U*=3994; *P*=.002; *r*=0.14) than those who only completed the questionnaire.

**Table 1:** Characteristics of questionnaire respondents and interview participants.

Characteristics			Questionnaire respondents (n=424)		Interview participants (n=30)
Age (y), mean (SD)			40.8 (9.9)		43 (9)
Satisfaction (0 to 10), mean (SD)			8.0 (2.0)		8 (2)
Adherence (%), mean (SD)			74.5 (29.4)		88 (23)
**Biological sex, n (%)**					
	Female	369 (88.1)		25 (83.3)	
	Male	50 (11.9)		5 (16.7)	
**Perceived contribution to self-awareness, n (%)**					
	Not at all	20 (4.8)		0 (0)	
	A little	93 (22.2)		6 (20)	
	Moderately	165 (39.5)		9 (30)	
	A lot	140 (33.5)		14 (46.7)	
**Psychological distress, n (%)**					
	Present	228 (54.3)		16 (53.3)	
	Absent	192 (45.7)		14 (46.7)	

### Correlation and Regression Analysis

For the study sample, a correlation analysis was conducted to examine relationships among the research variables (Table 2). Adherence was found to have small but significant positive associations with both satisfaction (*r*=0.19; *P*<.001) and perceived contribution to self-awareness (*r*=0.20; *P*<.001). In addition, a moderate positive correlation was observed between satisfaction and perceived contribution to self-awareness (*r*=0.37; *P*<.001). Regarding psychological distress, small negative correlations emerged with age (*r*=−0.18; *P*<.001) and satisfaction (*r*=−0.11; *P*=.03), suggesting that participants who experienced psychological distress tended to be younger and reported lower satisfaction with the app. No significant association was found between psychological distress and adherence (*P*=.08).

**Table 2:** Correlation analysis (Pearson r and two-tailed *P* value) among research variables.

Variable			BS^a^		Age		PD^b^		Adherence		Satisfaction		PCSA^c^
**BS**													
	*r*	1		0.013		−0.011		−0.020		−0.027		−0.001	
	*P* value	—^d^		.80		.83		.69		.58		.99	
**Age**													
	r	0.013		1		−0.175		−0.049		0.056		0.085	
	*P* value	.80		—		<.001		.32		.26		.09	
**PD**													
	*r*	−0.011		−0.175		1		0.086		−0.107		0.001	
	*P* value	.83		<.001		—		.08		.03		.98	
**Adherence**													
	*r*	−0.020		−0.049		0.086		1		0.191		0.201	
	*P* value	.69		.32		.08		—		<.001		<.001	
**Satisfaction**													
	*r*	−0.027		0.056		−0.107		0.191		1		0.365	
	*P* value	.58		.26		.03		<.001		—		<.001	
**PCSA**													
	*r*	−0.001		0.085		0.001		0.201		0.365		1	
	*P* value	.99		.09		.98		<.001		<.001		—	

^a^BS: biological sex.

^b^PD: psychological distress.

^c^PCSA: perceived contribution to self-awareness.

^d^Not applicable.

A hierarchical multiple regression analysis was conducted predicting adherence with age and biological sex entered first, satisfaction with the app, perceived contribution to self-awareness, and presence of psychological distress entered second (Table 3). The first model with age and biological sex was not statistically significant (*F*_2,404_=0.79; *P*=.46). The second model, which included all predictors, was statistically significant (*F*_5,401_=6.59; *P*<.001), accounting for 7.6% of the variance in adherence. Specifically, satisfaction (β=.16; *t*_401_=3.14; *P*=.002) and perceived contribution to self-awareness (=0.15; *t*_401_=2.89; *P*=.004) were significantly associated with adherence, indicating that individuals with higher satisfaction or perceived contribution to self-awareness scores had higher adherence. However, the psychological distress was not a statistically significant predictor of adherence (*P*=.07).

**Table 3:** Multiple hierarchical regressions analysis of adherence.

Models		*B*	β (SE; 95% CI)	*t* test (*df*)	*P* value	*R^2^*	
**Model 1**							.004
	Biological sex	−1.76	−.02 (4.53; −10.66 to 7.14)	−0.39 (404)	.77		
	Age	−0.17	−.06 (0.15; −0.46 to 0.12)	−1.18 (404)	.24		
**Model 2**							.076
	Satisfaction	2.49	.16 (0.79; 0.93 to 4.05)	3.14 (401)	.002		
	PCSA^a^	4.99	.15 (1.73; 1.58 to 8.40)	2.89 (401)	.004		
	PD^b^	5.25	.09 (2.87; −0.39 to 10.88)	1.83 (401)	.07		

^a^PCSA: perceived contribution to self-awareness.

^b^PD: psychological distress.

### User Experience Themes

#### Overview

Interviewees’ characteristics are provided in Table 1. From the thematic analysis, 3 main themes emerged (Textbox 1). The first theme regrouped instances in which participants described psychological self-monitoring as an introspective practice, imbued with reports of self-awareness and self-care. The second theme characterized the app as practical but not a one-size-fits-all, and presented how different app properties influenced user experience. The last theme regrouped potential enhancements that the participants mentioned to improve their experience.

**Thematic analysis of user experience interviews.**

**Introspective practice**

- Self-awareness: Taking time to pause; focusing; acknowledging; reacting to alerts
- Self-care: Being more attentive; talking to support system; consulting primary care physician; practicing self-compassion; problem-solving

**Simple app but not a one-size-fits-all**

- Practical use: Ease of use; time efficiency; portability
- Kinds of questions: Clarity; repetitiveness; range
- Presence of notifications: Incentive; bothersome
- Display of resources: Relevant; rarely used

**Potential enhancements**

- Feedback: Weekly overall assessment; relativity to other users; progress tracking
- Writing space: For reflection; for context
- Beyond self-monitoring: Psychological tools; discussion forum; maintaining simplicity

#### Introspective Practice

Participants reported a rather unanimous view of psychological self-monitoring. Most participants viewed completing the weekly questionnaires as a moment for reflection and self-awareness, allowing them to pause and consider their well-being. This opportune time allowed them to take a step back to focus on their state, allowing its recognition. Some participants added that psychological self-monitoring enhanced awareness of psychological fluctuations over the weeks:

When I was answering the questions, it was [helpful] because I had the time to ask myself the question and to question myself: am I stressed? Are there things that get to me more than before?... It seemed to make me aware of what was really going on around me, and it gave me an indicator of “am I more stressed than usual? Is it getting to me? How do I see things around me? Are things going well, or not so well?”

Sam

I found that [using the app] had benefits, in the sense that it helped us to make certain realizations, that it helped us to take a moment to stop when it’s not always something we think about doing.

Maria

Half of the participants in this subsample received a message alerting of a significant psychological distress, leading to varied reactions. Some participants were not surprised by the warning. Feeling it aligned with their internal state, they considered the psychological self-monitoring app reflected their experience:

[The distress warning] didn’t surprise me because I know myself well and I’m really familiar with mental health problems, so I was able to see what was going on.

Maria

It just echoes something I was feeling, but having an external message perhaps helped me to confirm that feeling a bit. Logically, it’s not just me imagining things that aren’t going well and I do have the right to say to myself that there’s something wrong and that it’s not just me imagining things.... In any case, this message confirms that I have to be “easy” on myself. That’s what it’s saying.

Mark

Interestingly, a participant with lasting distress reported her frustration to be repeatedly “told again” about it and recommended less frequent self-monitoring:

I don’t remember how many weeks it lasted..., but I knew I wasn’t doing well so I was still going to be told that I wasn’t doing well. [The distress warning] is still going to come out....You know, I don’t need to be told again, I know it’s about the same level. But I think, for someone who doesn’t know or who wants to assess or who’s in between, I think it’s important to do it often.

Nicole

However, others were caught off guard. Initial reactions of surprise, irritation, anger, or denial were reported in the interviews, as demonstrated in the subsequent quotes. Acknowledging the distress warning as a reflection of their questionnaire responses was reported as an important step toward self-awareness and self-care practices:

[Receiving the distress warning] was still a surprise, it really annoyed me, I always had warnings...Except it was because I was in denial!...it’s just that it was spitting all my distress and confusion and despair right back in my face....at first, I was genuinely irritated. I was getting tired of it; it was getting on my nerves. Later, at some point during the summer when I was filling in the damn questions, I was like “why do I feel this way? why do I feel like bawling?” Then, at some point, I was answering the questions and I was so uncomfortable I had tears. Then, I was like “OK, let’s stop burying our heads in the sand!”

Jude

I was actually surprised, because I wasn’t expecting [to receive the distress warning]. I didn’t really think anything was wrong.... It really made me react. It got to me....I found [the psychological self-monitoring app] helpful in the end. It was a help. Maybe it prevented me from falling into depression or whatever.

Riley

For some participants, the distress warning was seen as a “wake-up call” (Victoria) or “a kick in the butt” (Charlie) that prompted them to take actions, such as consulting their physician (Charlie). Other actions taken after receiving the warning included being more attentive to one’s condition, being kinder to oneself, seeking and adopting concrete solutions for individual stressors, etc:

[The distress warning] led me to call my psychiatrist and talk about it...When it asked the famous question “Would you be better off dead?” I answered “yes.” But I answered the question. I was honest with myself. I was surprised by my message, but I tell myself it’s a reflection of my answers.

Megan

However, participants who did not receive the distress warning also reported adopting changes prompted by the psychological self-monitoring practice. Actions included reducing media exposure; talking with family, friends, and colleagues about their mental health; asking for help from their manager; and seeking work-life balance. This mobilization was often propelled by their reaction to their responses when filling out the questionnaires:

When I say more negative things in the app, I try to verbalize them a little more with my family...or I try to change my way of seeing things too...I try to put things into perspective a little, and tell myself that how I felt was normal.

Sophia

A small proportion of participants specified that they might have adopted those changes without psychological self-monitoring. However, they presumed that they would have delayed action or waited for their condition to worsen before acting:

[Self-monitoring] actually made it a bit easier to take the decision to say “I’ve got this job to do, but it won’t be at all costs,” to give myself limits. [Without the app], maybe it would have taken longer to set up, maybe it would have come later. Maybe it would have taken on proportions that would have been a bit more troublesome.

Mark

I saw that things were starting to go downhill more and more, so I stopped listening to the press briefings....I even deleted my [social media account] just to give you an idea....Otherwise, I started doing more yoga, to switch things up while I waited for the pool to reopen. [Without the app], maybe it would have taken longer for me to realize what was going on.

Julia

Interestingly, about half of the interviewees viewed psychological self-monitoring itself as a self-care practice, considering the app as an effective psychological self-help tool. As demonstrated in the following quotes, some even integrated it into their routine, and it became a habit to the point of waiting for the weekly notification:

I thought that [psychological self-monitoring] was a moment for me, I always did it at the same time, after reading the newspaper on Saturday, and it was a bit of a retrospective of my week, part of my little ritual of taking care of myself, after my newspaper, my coffee, and then I wondered how I was doing.

Mika

It’s so useful. I found it interesting because every week, you were practically waiting for the app to tell you “you have a new survey to do.” It allowed me to keep up to date with where I was...I thought it was a great, relevant tool, [especially] for someone like me who has generalized anxiety disorder.

Megan

It allows me, even now [post-participation], to take more time, to self-monitor, to look at how I’m doing, to stop and say to myself “well, is this good? Or am I going the wrong way?”

David

A minority of participants reported that psychological self-monitoring did not have a significant impact on the way they managed their well-being. Notably, they considered that they were already managing well on their own or that no particular actions were needed:

I’m not sure [the app influenced my actions], maybe like 10%-5%....I’m quickly looking for solutions and thinking about how I can think differently.

Clara

[When I got the distress warning] I wasn’t really surprised...But I didn’t feel that I necessarily needed help, you know, I read a lot about that and maybe that’s why I didn’t seek specific help, I didn’t feel that it was that problematic in relation to work.

Patricia

#### A Practical App but Not a One-Size-Fits-All

Overall, participants found the psychological self-monitoring app practical, which seems to have contributed to their satisfaction. They appreciated that the app was simple, easy to use, and not time-consuming (approximately 5 minutes on average). In addition, the app’s accessibility on smartphones contributed to its portability and practicality:

I often did it at work between two things. It paged me, I did it straight away. I found it practical. It’s good because it doesn’t take long to do.

Charlie

[The app] was friendly, easy, I liked it, I had a reminder, “oh yes, I have to answer.” Again, it’s fast. The fact that it’s an app, we all agree, it’s on a different level compared to the paper questionnaires, we can agree that it was a lot easier.

Sara

Few participants reported technical issues, such as glitches and the absence of notifications, which negatively affected their experience and adherence. Very few contacted the research coordinator for help:

At a certain point, it just sort of fell through. I don’t know why, I had some sort of technical problem and it didn’t work. And after that, I kind of lost interest and disengaged.

William

How participants perceived the questions seemed to have influenced their experience and overall satisfaction. First, most of the participants found the questions clear and direct. The use of Likert scales made psychological self-monitoring easy and fast, with very few participants reporting that the questionnaires were long and that it was sometimes hard to choose between 2 scales:

The highlights [of the app]? The questions. I mean, they were clear. It wasn’t complicated.

Julia

What I liked about the app was that it was easy to use. The questions were simple. Sometimes I’d get stuck between two answers. I said to myself that it’s normal because they’re not development questions....In general, the questions were quite explicit.

Frederick

Second, users had to answer a wide range of questions. Some participants reported finding them extensive because personal and professional aspects were included:

[The questions] were so broad, it seemed to cover every area of mental health you could experience. I was really positively surprised.... [My expectations were] exceeded even, because it was all subjects that I didn’t expect that I might have difficulties with in my life,...it’s things that you don’t think about, it’s so banal, but it’s true that it was affected by COVID.

Sophia

I liked the app and the questionnaires because there was a lot of professional and personal portions to see what affected me the most.

Sabrina

However, a significant portion of participants found them too centered on either psychological symptoms or COVID-19. For instance, a participant expressed less interest in questions about COVID-19 exposure from patients. Three participants (David, Mika, and Isabelle) explicitly mentioned the desire to skip the questions they perceived as less relevant to them, such as PTS symptoms assessed in the PTSD Checklist for DSM-5:

I work in a hospital, but I’m in a field where I’m not close to patients. So there were questions about [exposure to COVID-19] where I felt a little less concerned, even though yes, the pandemic affected my work very, very directly...It’s as if there were questions, I would have liked to skip sometimes, because I thought “well, it’s not for me.”

Axel

[As improvements,] if it would have been possible to skip the PTS thing since it never applied to me. I could have eliminated a third of the questions because, unless you’ve been through a trauma while you’re answering, I mean, it’s quite surprising that this aspect changes when you haven’t been through a trauma.

Isabelle

Nevertheless, few participants mentioned a sense of comfort knowing that the app was tailored for HCWs’ well-being, particularly during times of crisis. They invited their colleagues to participate in the research project, showed interest in using the psychological self-monitoring app for more than 12 weeks, or suggested to make such apps available for all HCWs:

I found [the app] recomforting. It was like someone was...thinking about taking care of my well-being.

Remy

It went really well. It was very positive and I’d be happy to use it again if there were ever another study like that with this app.

Riley

I think that [the psychological self-monitoring app] is something that needs to be deployed among HCW, because I don’t think we realize how much more distressed we [actually] are...I could even be a spokesperson and say, “Look, it bothered me so much at the beginning...” I found it tiring, but it was salutary for me. I wasn’t really expecting it, but that’s what it was.

Jude

The repetitiveness of the weekly questions generated divided opinions. On one side, it was redundant, and some participants admitted paying less attention or being more passive when filling out the questionnaires. On the other side, it was predictable. Some participants appreciated knowing what would be asked of them in advance and revealed paying more attention to their state during the week. Regardless, the repetitiveness allowed some to identify trends and changes over the 12-week period:

Since it was always the same questions from week to week, I found that it made things easier once you got used to the questions.... I’m someone who’s introspective and self-observant, so in the week between answering the questionnaires, I paid a bit more attention....I knew what to pay more attention to.

Maria

I found the questionnaire relatively long, and at a certain point, you come to know the questions and generally, yes, there’s variability in some places, but there are other questions that haven’t varied all that much.

Mika

I thought the questions would change over time. But I found that they were always pretty much the same questions. At some point, not that you want to, but it becomes automatic when you answer the question. You think less about it because it’s the same questions....But still, sometimes yes it was the same question, but sometimes I didn’t give the same answer either, and it made me think. You know, what did I do this week that made me more tired, or more stressed? It gave me time to stop and think.

David

The notification reminding participants to complete the psychological self-monitoring seemed to have impacted their satisfaction and adherence. As expected, notifications served as an incentive; participants who did not receive any notification reported either forgetting to complete their monitoring or losing interest in the app:

The good thing was that even if sometimes I forgot to take part in the study during the day...the app would remind me that I hadn’t completed it. I found that interesting. I found it practical.

Frederick

At the same time, a few participants expressed dissatisfaction with the timing of notifications. Participants found that those sent at inconvenient hours, such as during sleep, were bothersome and preferred more control over notification timing:

The only annoyance I’ve had with the app is the notifications. I turned off the app’s notifications because it was ringing at midnight or 5am on days off. I was a bit at a loss.

Audrey

Regarding help resources displayed upon the completion of the questionnaires, most participants reported not using them despite finding them relevant. Furthermore, a minority of participants reported not noticing those resources, and a few were confident they were not displayed, possibly due to technical issues:

I took [screenshot of the resources] and I really took it as a helping hand, saying “we’re really here, whatever happens...”But it was really circumstances that made me turn more to my parents, to my partner, to friends.

Sabrina

#### Potential Enhancements

When discussing their expectations and recommendations for the psychological self-monitoring app, participants mentioned several desired features not currently offered. First, many expressed a desire for feedback after completing the questionnaires, suggesting that a form of result, such as an overall assessment, would enhance their experience. Some suggested a score or a graphic to help them track their progress through the weeks. In the following extract, a participant mentioned her expectation to see an overall assessment of other users:

A comment on what more could have been done, for example, is to have a view of the longitudinal evolution. From one week to the next, is it “better”? Is it less good? In other words, having feedbacks.

Mark

What I also expected was to have an overall picture, my overall picture at the end, over the course of the weeks. Like a small graph, it’s going well, it’s not going well, something like that. Then, I also expected to have an overall portrait of the people who participated in general so I could have a comparison too. Are we all in the same state of mind? Or is it just that...you know, sometimes you feel a bit alone out there, wondering if you’re...normal.

Alex

As improvements, a visual or an awareness for people to see, let’s say, the percentage of COVID or the percentage of how people actually assess their stress. It’s always the same questions but you can’t really see the results...see the survey pulse. It would have been fun to see how people felt and whether we’re part of the people who are on average more stressed, less stressed, etc.

Sam

Second, a few participants mentioned an interest for a writing space within the app. Some envisioned using it to provide context for their answers, as they felt certain responses were influenced by unrelated events and wished to add comments. Others wanted it as a journaling space to reflect on their feelings and thoughts while completing the questionnaires:

It’s easy with answer choices, but each time, I had a comment that I would have added. I don’t know how to say this...In addition to the answer choices, I would have liked if there was a little box, 200 words, because in the end I was often left wanting more, thinking “yes but.”

Sara

Perhaps I was expecting to have spaces for answers and comments, as if to clarify my thoughts? It wasn’t in the app.

Axel

Finally, some participants expressed a desire for features that extend beyond self-monitoring. For example, a few participants suggested integrating psychological tools within the app, such as the informational sheets sent by email and tailored coping strategies. One participant [William] also mentioned the possibility of chatting with other users or counselors:

Maybe the app could suggest things, maybe that’s why there’s no colour, why it’s more neutral.

Clara

[To enhance the app] if, say, I’d had a bereavement because of COVID, if I’d been told, on the basis of what I’d answered in the questionnaire, “we can see that you’ve experienced such and such a thing this week, try such and such a thing.”

Axel

Perhaps there could have been tools for sharing with others who are in the same situation as us....You know, when you’re told “be careful, it’s red,” maybe there could be exchange groups or chat rooms with other people like us, with counselors. It could have been something great.

William

However, many participants appreciated the app’s simplicity and recommended maintaining that aspect:

[To enhance the app], I don’t really know, because it’s important that it’s not too long. I’m not sure that adding things is relevant.

Patricia

No [suggestions], I’d keep it that way. What I liked was that it was short. That too was appreciated. It didn’t take an hour. I liked that, but no, I don’t see what could be different.

Emily

## Discussion

### Principal Findings

#### Overview

This mixed methods study aimed to explore Canadian HCWs’ experience with a psychological self-monitoring app. To examine whenever satisfaction, perceived contribution to self-awareness, and experience of psychological distress influenced users’ adherence, a multiple hierarchical regression analysis was performed on quantitative data while adjusting for age and biological sex. To further explore user experience, thematic analysis was performed on qualitative data.

Over 12 weeks, the average adherence rate to the psychological self-monitoring app was 74.5%. This can be characterized as high considering that only 0.5% to 28.6% of research participants using self-help apps or programs for depression and anxiety sustain their use after 6 weeks [29,66]. Statistical analysis suggested that approximately 7.6% of adherence’s variation could be explained by satisfaction with the app and perceived contribution to self-awareness. Thematic analysis of qualitative data offered a more profound exploration of participants’ experience. Mixed methods findings are discussed following the key areas Chan and Honey [67] identified in their integrative review on users’ perceptions: satisfaction, ease of use, helpfulness, technical and perceived issues, and improvements. Then, the specific case of psychological distress is discussed.

#### Satisfaction

The high satisfaction rate of 80% was unexpected given that the psychological self-monitoring app was primarily intended for data collection. Interface design is known to significantly impact satisfaction and adherence [68], with visual appeal [69] and personalization [70] enhancing user experience. The Avicenna app used for psychological self-monitoring offers limited design options, but the qualitative component of this study may help explain the discrepancy between our results and prior research. Interviewees clarified the unexpectedly high satisfaction rate, emphasizing the app’s practicality, simplicity, and time efficiency. Unlike in the study by Alqahtani and Orji [71], no one criticized the basic interface. This suggests that users are more likely to adhere to a psychological self-monitoring app they perceive as convenient, consistent with findings from other studies [41,72]. Thus, even a very basic psychological self-monitoring app can offer a satisfactory user experience.

It could also be explained by the way satisfaction was conceptualized. In this study, it was defined broadly as an overall reflection of users’ appreciation of their experience with the app. When measured, satisfaction was explained to participants with nonexhaustive examples (“eg, ease of installation, friendliness, etc”). Due to this large definition, participants may have prioritized assessing ease of use over design. As noted by Ng et al [73], satisfaction often overlaps with other user engagement indicators such as usability and acceptability, and further research is needed to clarify its definition in the context of mental health apps.

#### Ease of Use

Furthermore, interviews revealed that a key facilitator of app’s adherence and satisfactory experience was its ease of use. Psychological self-monitoring required users to answer questions that were perceived as clear, straightforward, and requiring minimal efforts. This could corroborate with other studies suggesting that users prefer apps that avoid cognitive overload [33]. Given that participants were HCWs in a health crisis, a simple and intuitive app may be suitable for this population, providing a straightforward experience under stressful conditions.

#### Helpfulness

In parallel, satisfaction rates may have been influenced by users’ perceptions of the app’s usefulness or helpfulness. This study hypothesized that the usefulness of a psychological self-monitoring app lies in its ability to enhance emotional self-awareness and found that perceived contribution to self-awareness was positively associated with both satisfaction and adherence, corroborating previous reviews [32,41]. Notably, 305 out of 424 respondents (71.9%) perceived that the app contributed at least moderately to improving their emotional self-awareness. This is illustrated with how interviewees characterized their experience as introspective. Completing questionnaires about COVID-19 exposure and its impacts on psychological state seemed to have heightened participants’ attention to their well-being. This finding is significant for HCWs, who are expected to prioritize their patients’ needs over their own, particularly in time of health crisis.

This result is particularly interesting for interviewees experiencing psychological distress. Some were surprised when the app alerted of significant distress, suggesting varying levels of emotional self-awareness before monitoring. Those surprised likely had lower initial emotional self-awareness and found the app valuable for enhancing it, possibly leading to higher perceived contribution to self-awareness, satisfaction, and adherence. Conversely, participants already aware of their distress may not have felt that the app significantly improved their self-awareness, likely impacting the perceived contribution to self-awareness, satisfaction, and adherence. Overall, initial emotional self-awareness might influence user experience and adherence, indicating the need for further research in this area.

In addition, some interviewees integrated the use of the app into their weekly routine. Developing a habit of psychological self-monitoring can not only enhance users’ adherence [33,72] but may also persist after discontinuing app use [74]. Longitudinal studies are required to assess the potential long-lasting effects of psychological self-monitoring on users’ practices.

Furthermore, many participants reported taking steps to maintain or enhance their well-being, regardless of whether they were experiencing distress. This reinforces the idea that self-awareness is crucial for adopting coping strategies [17] and that it can mediate the positive association between psychological self-monitoring and well-being [12-16]. Notably, some interviewees hinted that psychological self-monitoring may have facilitated earlier emotional self-awareness and mobilization, potentially preventing psychological distress among HCWs.

#### Technical and Perceived Issues

The most frequently reported barrier to app use was the absence of reminders or their inconvenient timing. Reports indicating that reminders positively impact adherence, while their absence negatively affects it, align with a systematic review [70]. In addition, few interviewees were bothered by reminders displayed at inconvenient times. This corroborates previous findings suggesting detrimental effects of reminders sent at inconvenient times or places on app adherence [33].

Some interviewees reported finding the psychological self-monitoring questions redundant or too centered on a certain aspect. Those criticisms may hint at users’ diverse monitoring needs. The app did not offer the option to customize questions, limiting users’ ability to tailor their psychological self-monitoring experience to their individual preferences. Considering the critical role of app personalization [70,75], allowing users to select the questionnaires they find most pertinent to monitor may foster a more relevant experience. Thereon, the app admittedly did not adopt a user-centered approach. The fact that some HCWs expressed comfort, knowing the app was deployed out of concern for their well-being, suggests that users may value apps specifically tailored to them.

#### Improvements

Reported suggestions to improve the app consisted of adding features to enhance psychological self-monitoring and supposedly self-awareness. A common recommendation involved incorporating feedback mechanisms, such as displaying results or summaries within the app. Many interviewees expected some form of feedback or visual outcome. This desire aligns with existing evidence highlighting the perceived usefulness [66,76] and effectiveness [77] of feedback in mental health apps, as well as users’ interest in tracking progress or trends in their behavior through visual or numerical summaries [33]. More specifically, providing users with their scores on assessment tools and allowing comparisons with aggregated results from other users could further enhance self-awareness and engagement, as suggested in studies of apps targeting anxiety and depression [75,78,79].

In addition, few interviewees suggested integrating a writing space either to add contextual information or to journal about their experience. Thereon, a study suggested that a mood-tracking and journaling app can enhance introspection [80], and another study found that journaling was associated with improvements in psychological well-being of users with a predisposition for self-reflection [81].

While a proportion of interviewees liked the app for its simplicity, some suggested including more complex features and components within the app, namely educational content and discussion forums. These recommendations suggest unmet needs for psychological information and social connections, which can negatively impact user experience and adherence. Indeed, health information and social opportunities were found to increase users’ intention to engage with well-being apps [33]. This suggests that an app offering psychological self-monitoring as its only feature may not be suitable for users who have needs beyond an active monitoring of their psychological well-being. Integrating tools to help users manage psychological well-being, along with personalized strategy recommendations based on individual distress profiles, could improve the perceived relevance of the app. Prior research has shown that tailoring interventions to users’ specific profiles, rather than offering a wide range of generic strategies, tends to increase engagement and perceived usefulness [82].

As previously reported, participants also mentioned a desire to choose the questions they are asked, the timing they receive the reminders, and the frequency of their self-monitoring. As hinted in other studies [71,75,83], this result highlights the importance of not overlooking personalization opportunities, even in basic self-monitoring apps. Promoting user interaction with the app by offering options to customize certain components may help reduce perceptions of repetitiveness or inconvenience and increase alignment with individual preferences, ultimately supporting greater adherence [70].

#### Experience of Psychological Distress

Correlation analysis suggested an association between age and psychological distress, with younger participants reporting more distress than older participants. This result aligns with the research by Park et al [84], in which younger adults reported greater distress and less social support, mindfulness, and emotion regulation skills than older adults during the pandemic. In the same vein, an observational study used socioemotional selectivity theory, suggesting that older adults are motivated to optimize their emotional experiences in the limited life that they have left [85], to explain similar result in their 29-wave longitudinal survey [86]. These findings underscore the vulnerability of younger HCWs and the importance of ensuring that support strategies are accessible and relevant to their specific needs.

Although the presence of psychological distress was negatively associated with satisfaction and that satisfaction was positively associated with adherence, the correlation between psychological distress and adherence was not statistically significant (*P*=.08), suggesting a potential trend that warrants further investigation. Previous studies found conflicting results regarding psychological distress and adherence to mental health apps [34,42-47]. The hypothesis that users in distress would adhere more to a psychological self-monitoring app as they might feel a greater need to thoroughly monitor their state was infirmed. The expectation-confirmation model [37] and the technology acceptance model [78] are 2 complementary models that may help explain these results.

According to the first one, users’ expectations and needs play a crucial role in their satisfaction and subsequent adherence. Some participants were satisfied with the app’s simple features, as it met or exceeded their expectations, positively influencing satisfaction and adherence. Others might have had higher expectations and needs that the basic psychological self-monitoring app did not fulfill, leading to lower satisfaction and reduced adherence. This could be particularly the case of distressed users, who might have expected features and components in the app that could help alleviate their distress, such as the recommended inclusion of psychoeducational materials and personalized strategies. This would align with prior research showing that psychological self-monitoring apps are most effective at reducing psychological distress when they include additional components, such as behavioral interventions and educational modules [12,25-28]. Hence, the app may have not met the heightened expectations of distressed users, leading to a lower satisfaction. Nevertheless, it seems that it was not detrimental to the point of affecting adherence. Indeed, some distressed users may have had other expectations that were met and that supported their adherence, despite an overall lower satisfaction. These insights highlight the importance of considering users’ various expectations and to clearly communicate to users what they can expect from an app to prevent deception.

The technology acceptance model further complements this perspective by emphasizing how 2 key factors, perceived ease of use and perceived usefulness, mediate the relation between external factors (eg, user characteristics and system features) and adherence [78]. In this study, although the app may have been perceived as easy to use, its perceived usefulness among participants experiencing psychological distress may have been limited, particularly those who were self-aware. Once again, these users were likely seeking concrete tools or strategies to manage their distress, which the app did not provide. As such, self-monitoring alone may not have been perceived as helpful by individuals in significant distress, possibly leading to reduced satisfaction and early discontinuation of use due to unmet needs. However, adherence may be influenced by other factors, such as users’ intrinsic motivation, which might not be directly impacted by psychological distress.

Moreover, results suggest that users, whenever in distress or not, can experience positive outcome from a basic psychological self-monitoring app. While psychological distress can be associated with less satisfaction, it does not necessarily deter users from adhering to the app or perceiving it as beneficial. This suggests that even if the app does not fully meet the expectations and needs of users, certain aspects may still provide value, leading to continued use and perceived usefulness. Future research, notably randomized controlled trails, is needed to rigorously assess the impact of minimalist psychological self-monitoring apps on multiple well-being outcomes.

### Clinical Implications

These findings have clinical implications, suggesting that psychological self-monitoring could benefit individuals struggling with self-awareness by enhancing emotional self-awareness and encouraging self-care, and facilitating early detection of distress to prevent further aggravation. Organizations could propose psychological self-monitoring programs to support well-being, particularly in professions at high risk of burnout and turnover. Although this study focused on Canadian HCWs’ experience during the COVID-19 pandemic, research, including randomized controlled trials, is needed to assess psychological self-monitoring app effects. However, findings suggest that the app helped some users open up to their support system about their mental health, including their managers and colleagues. Mental health clinicians could incorporate psychological self-monitoring apps into their practice as part of a comprehensive plan, using collected data to explore clients’ symptoms and coping strategies. Clinicians should focus on offering personalized feedback and educational materials.

### Limitations and Future Studies

Several limitations should be considered when interpreting the present findings, and external validity must be approached with caution. First, participation bias and convenience sampling may have influenced both the quantitative and qualitative results. Although all 831 app users were solicited to fill the postparticipation questionnaire, only 424 (51%) responded, and 149 (17.9%) agreed to be interviewed. Comparative analyses between participants and nonparticipants were conducted posteriori and suggested that higher participation in the research study was associated with greater adherence, with interviewees showing the highest adherence. This trend may indicate that more engaged or satisfied users were overrepresented, potentially leading to an overestimation of adherence and other positive outcomes such as user experience. While the mixed methods approach is a strength, a sequential design could have provided deeper insights, particularly for users with lower adherence, satisfaction, or those experiencing psychological distress.

Second, regarding sample characteristics, a predominance of biologically female participants was observed, representing 88.1% (369/419) of the quantitative sample and 83% (25/30) of the qualitative sample. However, this heterogeneity is consistent with the health care workforce population in Quebec where a similar sex disproportion has been reported in 2023 [87].

Third, the generalizability of the findings to other regions should be considered cautiously. Crisis support resources offered in the app were specific to users’ institutions and pandemic-related initiatives. Nevertheless, the psychological distress levels observed in the sample are comparable to those reported in meta-analyses of HCWs’ mental health during the COVID-19 pandemic, and the instruments used to assess distress were consistent with those used in international studies [7,8,48,49]. It is therefore reasonable to believe that the findings could be similar for other HCWs in industrialized countries during the COVID-19 pandemic.

Finally, psychological self-monitoring was assessed through self-reports collected on a weekly basis, introducing a potential recall bias. To enhance accuracy, ecological momentary assessment typically consists of 6 assessments per day [88]. However, data suggest that users may have different preferences and offering a customizable self-monitoring frequency could represent a more acceptable alternative, potentially improving user experience and adherence.

This study findings reinforce the idea that a one-size-fits-all approach limits the potential of apps and digital interventions, even when users perceive them as easy to use and helpful. Notably, satisfaction and perceived contribution to self-awareness accounted for less than 8% of the variation in adherence. In the context of psychological self-monitoring for HCWs, additional app characteristics and features need to be explored to better meet users’ diverse needs. Future research should incorporate available design recommendations [68,89] while paying close attention to the specific needs and preferences reported by HCWs in this study. More broadly, the COVID-19 pandemic has highlighted the urgent need for proactive programs to support HCWs during crises. Although robust evidence for the effectiveness of psychosocial interventions remains limited [90], the findings suggest potential benefits of basic psychological self-monitoring apps. Further research is warranted to evaluate their impact on psychological well-being and to identify optimal design features that maximize adherence, satisfaction, and mental health outcomes.

### Conclusions

A simple psychological self-monitoring app could be an interesting tool for individuals who want to improve their emotional self-awareness and proactively adopt self-care practices. This study presented how HCWs in a health crisis may benefit from such apps. Some of the adherence to the app could be explained by how satisfied users were and how usefully they thought the app contributed to enhancing their self-awareness. Complementary features, such as access to feedback and psychological tools, may enhance the user experience. Simplicity, ease of use, tailoring, and customization options should be considered when developing psychological self-monitoring apps.

## Appendix

Multimedia Appendix 1 Questionnaires on COVID-19 exposure, social support, and quality of life.

Multimedia Appendix 2 Postparticipation questionnaire.

Multimedia Appendix 3 Interview grid-participation questionnaire.

Multimedia Appendix 4 Characteristics of the participants in different phases of the research.

## Abbreviations

HCW: health care worker
PHQ-9: Patient Health Questionnaire-9
PTS: posttraumatic stress
