# Supplementary material for: Health Care Workers’ Experience With a Psychological Self-Monitoring App During the COVID-19 Pandemic: Mixed Methods Study
Source: JMIR Mhealth Uhealth. 2025 Aug 7;13:e70412. doi: 10.2196/70412 (PMC12371282; doi:10.2196/70412)
Supplement: Multimedia Appendix 1 [file mhealth_v13i1e70412_app1.docx]

## Multimedia Appendix 1

### Questionnaire on COVID-19 exposure

1. Tick all situations of contact with a person in whom a COVID-19 infection is suspected or confirmed in the last week:

Patient Colleague Fellow citizen Loved one None

1. Tick all situations of COVID-19 infection encountered in the last week:

| I am waiting for a test result | I have been confirmed positive for COVID-19 | I have a colleague who is positive for COVID-19 | I have a relative who is positive for COVID-19 | None of these situations |
| --- | --- | --- | --- | --- |

1. Tick all death situations encountered in the last week:

Patient Colleague Acquaintance Relative None

1. On a scale from 0 to 10, my level of fear of catching the virus or transmitting it to those close to me over the last week has been:

Nil Very high

0 1 2 3 4    5    6 7 8 9 10

1. On a scale from 0 to 10, my level of stress at work over the last week has been:

Nil Very high

0 1 2 3 4    5    6 7 8 9 10

1. The approximate amount of time I have spent catching up on COVID-19 news (TV, radio, internet, social media) in the last week is:

Almost never Sometimes Often Almost all the time

### Questionnaire on social support

1. Over the past 7 days, how available do you feel your loved ones were to offer you support?

Almost never Sometimes Often All the time

1. Over the past 7 days, how available do you feel your colleagues were to offer you support?

Almost never Sometimes Often All the time

1. Over the past 7 days, how available do you feel your organization was to offer you support?

Almost never Sometimes Often All the time

### Questionnaire on quality of life

1. Thinking about your life over the last 7 days, how would you rate your professional quality of life? Move the cursor to indicate your quality of life on a scale from 0 to 10.

Very poor Very good

0 1 2 3 4 5 6 7 8 9 10

1. Thinking about your life over the last 7 days, how would you rate your personal quality of life? Move the cursor to indicate your quality of life on a scale from 0 to 10.

Very poor Very good

0 1 2 3 4 5 6 7 8 9 10
