# Supplementary material for: Health Care Workers’ Experience With a Psychological Self-Monitoring App During the COVID-19 Pandemic: Mixed Methods Study
Source: JMIR Mhealth Uhealth. 2025 Aug 7;13:e70412. doi: 10.2196/70412 (PMC12371282; doi:10.2196/70412)
Supplement: Multimedia Appendix 2 [file mhealth_v13i1e70412_app2.docx]

## Multimedia Appendix 2

### Post-participation questionnaire

1. How satisfied were you with the mobile application you used to perform your self-monitoring for the project (e.g. ease of installing the application, user-friendliness of the application, etc.)?

0 1 2 3 4 5 6 7 8 9 10

1. To what extent has practicing self-monitoring of your psychological reactions through the app raised awareness or prompted reflection about your well-being?

Not at all A little Moderately A lot
