# Supplementary material for: Health Care Workers’ Experience With a Psychological Self-Monitoring App During the COVID-19 Pandemic: Mixed Methods Study
Source: JMIR Mhealth Uhealth. 2025 Aug 7;13:e70412. doi: 10.2196/70412 (PMC12371282; doi:10.2196/70412)
Supplement: Multimedia Appendix 3 [file mhealth_v13i1e70412_app3.docx]

## Multimedia Appendix 3

### Interview grid

| Coping strategies used | Generally speaking, how do you manage difficult days? |
| --- | --- |
| Opinion about the app | What did you think of the self-monitoring mobile app? |
| Coping strategies following self-monitoring | How do you feel about monitoring your own psychological state?  How have the app and self-monitoring influenced the management of your well-being? What effects do you think it has had on your actions and reflection?  Has self-monitoring prompted you to take action to improve your well-being? If so, how?  Would you have taken these actions without self-monitoring? |
| Reactions to distress warnings | Have you received any notifications about your psychological state? What did you think about it? What were your reactions? What did you do afterwards?  Are you aware of the support resources available to you? Have you consulted them? What prompted you to do so? What were the results? |
| Application | Do you use other wellness applications? If so, which ones and why?  Compared with these apps, what did you like about our self-monitoring app? What did you like less?  What were your expectations of the application? Were your expectations met?  What recommendations would you make for a new version? |
| Complementary | If a colleague were to ask you about our study, what would you say?  Is there anything else you'd like to add about psychological self-monitoring? About using a mobile app to improve well-being?  Do you have any further questions or answers? |
