# Supplementary material for: Health Care Workers’ Experience With a Psychological Self-Monitoring App During the COVID-19 Pandemic: Mixed Methods Study
Source: JMIR Mhealth Uhealth. 2025 Aug 7;13:e70412. doi: 10.2196/70412 (PMC12371282; doi:10.2196/70412)
Supplement: Multimedia Appendix 4 [file mhealth_v13i1e70412_app4.docx]

Table 1. Characteristics of participants who completed the questionnaire (n=424) and those who did not (n=407).

|  | | Completed questionnaire | | Group comparison | | |
| --- | --- | --- | --- | --- | --- | --- |
| Variables | | Yes | No | *U* | *χ^2^ (df)* | *P* value |
|  | |  |  |  |  |  |
| Age (years), mean (SD) | | 40.8 (9.9) | 39.0 (9.6) | 74310 | -^a^ | .018 |
| Adherence (%), mean (SD) | | 74.5 (29.4) | 52.0 (34.0) | 54513 | - | <.001 |
| **Biological sex, n (%)** | |  |  | - | 1.083(2) | .582 |
|  | Female | 369 (88.1) | 354 (88.3) |  |  |  |
|  | Male | 50 (11.9) | 46 (11.5) |  |  |  |
|  | Other | 0 | 1 (0.2) |  |  |  |
| **Psychological distress, n (%)** | |  |  | - | 0.087(1) | .768 |
|  | Present | 228 (53.8) | 223 (54.8) |  |  |  |
|  | Absent | 192 (46.2) | 184 (45.2) |  |  |  |

^a^Not applicable.

Table 2. Characteristics of participants who completed the questionnaire and were interviewed (n=30) and those who only completed the questionnaire (n=364).

| Variables | | Interviewed |  | Group comparison | | |
| --- | --- | --- | --- | --- | --- | --- |
|  | | Yes | No | *U* | *χ^2^ (df)* | *P* value |
|  | |  |  |  |  |  |
| Age (years), mean (SD) | | 42.9 (8.5) | 40.6 (10.0) | 4830 | -^a^ | .148 |
| Satisfaction (0-10), mean (SD) | | 8.3 (1.6) | 8.0(1.9) | 5286 | - | .565 |
| Adherence (%), mean (SD) | | 87.8 (22.9) | 73.5(29.6) | 3994 | - | .002 |
| **Biological sex, n (%)** | |  |  | - | 0.689(1) | .407 |
|  | Female | 25 (83.3) | 344(88.4) |  |  |  |
|  | Male | 5 (16.7) | 45(11.6) |  |  |  |
| **PCSA^b^, n (%)** | |  |  | - | 0.412(3) | .249 |
|  | Not at all | 0 (0.0) | 20(5.1) |  |  |  |
|  | A little | 6 (20.7) | 87(22.4) |  |  |  |
|  | Moderately | 9 (31.0) | 156(40.1) |  |  |  |
|  | A lot | 14 (48.3) | 126(32.4) |  |  |  |
| **Psychological distress, n (%)** | |  |  | - | 0.089(2) | .956 |
|  | Present | 16 (53.3) | 212(53.8) |  |  |  |
|  | Absent | 14 (46.7) | 182(46.2) |  |  |  |

^a^Not applicable.

^b^PCSA: perceived contribution to self-awareness.
